# Retraction: Reversal Effect of ST6GAL 1 on Multidrug Resistance in Human Leukemia by Regulating the PI3K/Akt Pathway and the Expression of P-gp and MRP1

**DOI:** 10.1371/journal.pone.0224304

**Published:** 2019-10-17

**Authors:** The PLOS ONE Editors

After publication of this article [1], concerns were raised about results reported in several figures.

Similarities were noted between western blot data reported in Figs 1B and 4A:

- Fig 1B ST6GalI lanes 4 (horizontally flipped) and 6 and Fig 4A p-Akt 308 lane 3
- Fig 1B ST6GalI lane 5 and Fig 4A p-Akt 308 lane 2
- Fig 1B ST6GalII lanes 4–6 and Fig 4A Akt lanes 1–3

In Fig 2B, there appears to be a vertical discontinuity after lane 2 of the GAPDH panel. The authors confirmed that they combined data from two blot fragments in this figure.

The authors provided image files and replication data in support of the above figures but these did not fully resolve the concerns.

In addition, several immunohistochemistry panels in in Figs 2E, 3E, and 5D that represent different experiments appear to show overlapping fields of the same data. (Panel numbers indicate columns in the figures, numbered from left to right.)

- Fig 2E ST6GalI panel 1 (lower right quadrant) appears similar to panel 2 (upper left quadrant).
- Fig 2E ST6GalI panel 2 is similar to Fig 5D PI3K p110α panel 1.
- For Fig 3E, the right edge of panel 1 and the left edge panel 2 are highly similar.
- There appears to be overlap between images shown in Fig 3E panel 3, Fig 5D p-Akt 308 panel 1, and Fig 5D p-Akt 473 panel 1.
- Similarities were noted in Fig 5D for p-Akt 473 panel 1 and Akt panels 2 and 3.
- In Fig 5D, the upper region of panel 2 for p-Akt 308 is similar to the lower region of panel 4 for Akt. The lower region of p-Akt 308 panel 2 appears to overlap with the upper region of NF-κB panel 4.
- In Fig 5D, similarities were noted between panel 2 for p-Akt 308 and panel 4 for NF-κB.
- In Fig 5D, there appears to be overlap between the images shown in p-Akt 473 panel 1, Akt panel 2, and Akt panel 3.

The authors commented that errors were made when preparing these figures and they provided updated versions in which the panels in question were replaced. However, the extent of duplications within and across figures calls into question the overall reliability of the results.

In light of the above concerns, the *PLOS ONE* Editors retract this article.

The corresponding author (JL) apologized for the issues with the published article and notified the journal that all authors agreed with the retraction. The other authors either could not be reached or did not respond directly.
